# Supplementary material for: Splice-Junction-Based Mapping of Alternative Isoforms in the Human Proteome
Source: Cell Rep. Author manuscript; Available in PMC 2020 Jan 15. (PMC6961840; doi:10.1016/j.celrep.2019.11.026)
Supplement: 2 [file NIHMS1546469-supplement-2.zip › DF1/PXD010154/Esophagus/Esophagus_21_RFWD2_QLEAQLEQIQK.pdf]

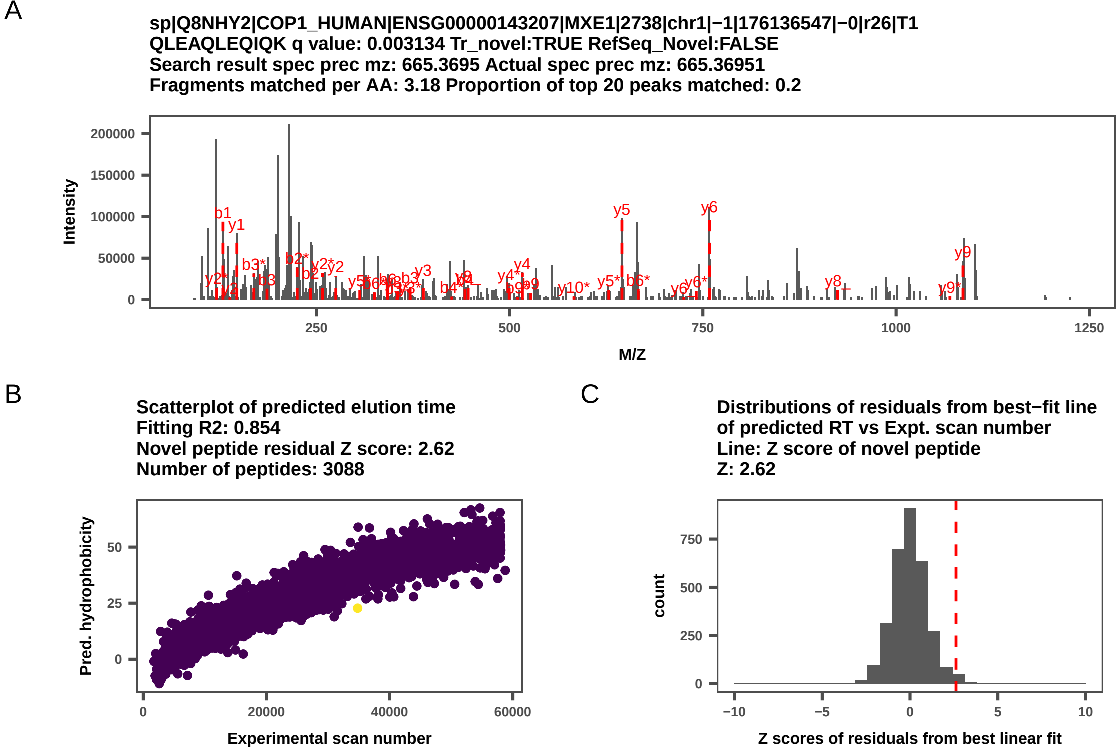
A
sp|Q8NHY2|COP1_HUMAN|ENSG00000143207|MXE1|2738|chr1|−1|176136547|−0|r26|T1
QLEAQLEQIQK q value: 0.003134 Tr_novel:TRUE RefSeq_Novel:FALSE
Search result spec prec mz: 665.3695 Actual spec prec mz: 665.36951
Fragments matched per AA: 3.18 Proportion of top 20 peaks matched: 0.2
Intensity
200000
150000
100000
50000
0
b1
y1
y5
y6
y9
y4
y8
M/Z
250
500
750
1000
1250
B
Scatterplot of predicted elution time
Fitting R2: 0.854
Novel peptide residual Z score: 2.62
Number of peptides: 3088
Pred. hydrophobicity
50
25
0
0
20000
40000
60000
Experimental scan number
C
Distributions of residuals from best−fit line
of predicted RT vs Expt. scan number
Line: Z score of novel peptide
Z: 2.62
count
750
500
250
0
−10
−5
0
5
10
Z scores of residuals from best linear fit
